# A robust immune-related gene pairs signature for predicting the overall survival of esophageal cancer

**DOI:** 10.1186/s12864-023-09496-x

**Published:** 2023-07-10

**Authors:** Wei Zheng, Gaofeng Fang, Qiao Huang, Dan Shi, Biao Xie

**Affiliations:** 1 Department of Cardiology, The First Affiliated Hospital of Chongqing Medical University, Chongqing, China; 2 Department of Nutrition and Food Hygiene, Chongqing Medical University, Chongqing, China; 3 Anatomy Teaching and Research Section, Basic department, Chongqing Medical and Pharmaceutical College, Chongqing, China; 4 Department of Biostatistics, Chongqing Medical University, Chongqing, China

**Keywords:** Esophageal cancer, Prognosis, Immune-related gene pairs, Bioinformatic analysis, Cells lines

## Abstract

**Background:**

Identifying reliable biomarkers could effectively predict esophagus carcinoma (EC) patients with poor prognosis. In this work, we constructed an immune-related gene pairs (IRGP) signature to evaluate the prognosis of EC.

**Results:**

The IRGP signature was trained by the TCGA cohort and validated by three GEO datasets, respectively. Cox regression model together with LASSO was applied to construct the overall survival (OS) associated IRGP. 21 IRGPs consisting of 38 immune-related genes were included in our signature, according to which patients were stratified into high- and low-risk groups. The results of Kaplan-Meier survival analyses indicated that high-risk EC patients had worse OS than low-risk group in the training set, meta-validation set and all independent validation datasets. After adjustment in multivariate Cox analyses, our signature continued to be an independent prognostic factor of EC and the signature-based nomogram could effectively predict the prognosis of EC sufferers. Besides, Gene Ontology analysis revealed this signature is related to immunity. ‘CIBERSORT’ analysis revealed the infiltration levels of plasma cells and activated CD4 memory T cells in two risk groups were significantly different. Ultimately, we validated the expression levels of six selected genes from IRGP index in KYSE-150 and KYSE-450.

**Conclusions:**

This IRGP signature could be applied to select EC patients with high mortality risk, thereby improving prospects for the treatment of EC.

**Supplementary Information:**

The online version contains supplementary material available at 10.1186/s12864-023-09496-x.

## Background

The new Esophageal cancer (EC) patients and deaths exceed 1,000,000 a year [1, 2]. The treatment for EC typically focuses on radiotherapy, chemotherapy, chemoradiotherapy endoscopic resection and surgery. Despite the advancements in diagnosis and treatment in recent years have improved the clinical outcomes of EC patients, the prognosis remained poor. The 5-year survival rate for EC is less than 20% [3]. Thus, identifying reliable prognostic biomarkers is necessary for the treatment of EC.

The immunity system is relevant to tumor formation and progression. The tumor microenvironment and immune cells participated in the initiation and deterioration of cancers [4, 5]. Immunotherapy has become an effective antitumor strategy of various cancers [6–8]. Represented by programmed death ligand-1, immune checkpoint inhibitors (ICIs) can restrain tumor growth and improve overall survival (OS) through inhibiting the immune escape [9, 10]. Esophageal tumor cells possess the high potential of immune escape because EC is characterized by high heterogeneity and high tumor mutational burden [11]. A few preclinical studies and clinical trials researchers have found that the ICIs pembrolizumab, nivolumab, camrelizumab and toripalimab can be used as a form of immunotherapy for EC [12–15]. However, immunotherapy for EC leads to mixed results. Indeed, several patients were shown to deteriorate sharply after receiving immunotherapy [16].

Recently, researchers have attempted to study the association between immune-related genes (IRGs) and the prognosis of EC [17–21]. For example, Wang and Li et al. ascertained the prognostic value of IRGs and infiltrating immune cells for EC patients [20, 21]. Some researchers used advanced computational tools. For example, Zheng et al. identified predictive and prognostic factors for esophageal squamous cell carcinoma based on Similarity Network Fusion and Consensus Clustering Analysis [22]. A study from China identified CLDN6 as a molecular biomarker in pan-cancer through multiple omics data integrative analysis [23]. Zhang et al. explored the role of YTH domain containing 2 in epigenetic modification and immune infiltration of pan-cancer [24]. Unsatisfactorily, none of these signatures could be translated to clinical application due to issues such as technical bias and biological heterogeneity of different platforms [25, 26]. To eliminate the disadvantage of normalization and scale transformation, researchers have developed a method on the basis of relative rank of gene expression value. This new method has been shown to produce robust signatures in prognostic stratification of various tumors [27–30]. In our study, an immune-related gene pairs (IRGP) signature was conducted to estimate the OS of EC.

## Materials and methods

### Public datasets and study design

The mRNA expression and corresponding clinicopathologic data of EC patients in the Cancer Genome Atlas (TCGA) and Gene Expression Omnibus (GEO) were acquired. The detail information of these cohorts is shown in Table S1. Altogether, four studies were enrolled, including TCGA cohort (n = 170), GSE13898 (n = 60), GSE19417 (n = 70) and GSE52625 (n = 179). Patients from TCGA cohort were allocated to the training set and all the patients from three GEO datasets were assigned to the meta-validation cohort. Patients without complete survival information and clinicopathologic information were excluded. In total, 479 patients were included in this study. 2483 IRGs were downloaded from ImmPort (https://immport.niaid.nih.gov) on 2022.05.01.

### Gene expression data processing

The function ‘normalizeBetweenArrays’ of R package ‘limma’ was first applied for the normalization of gene expression profiles, and then log-transformation was utilized for further normalization.

### Construction of predictive-related IRGP

Among the 2483 IRGs, only those existed on all platforms and with a relatively high median absolute deviation (MAD > 0.5) were selected. The IRGP score was obtained by contrasting the mRNA level of two paired genes in each sample. An IRGP score is assigned to 1 if the value of IRG1 was higher than that of IRG2, conversely, the IRGP score was 0 [20]. IRGP with steady value in datasets were removed. The left IRGP served as initial candidates for further analyses.

Prognostic IRGPs were chosen by the following steps. First, the association between each initial candidate IRGP and patients’ OS in the training dataset was assessed by the univariate Cox regression model. After rough filtration, the LASSO was further carried out to minimize overfitting. To improve the robustness of selected IRGPs, we randomly divided the training set into new training sets and test sets through a ratio of 2:1, and then duplicated this procedure 30 times. The LASSO was then used in the 30 training sets to choose those IRGPs with a frequency > 15. Ultimately, we adopted the multivariate Cox regression analysis to construct the IRGP-based model, which consisted of relevant IRGP index (IRGPI) and respective coefficients. The appropriate cut-off of IRGPI for the high- and low- risk groups was determined by receiver operating characteristic (ROC) curve at 3 years.

### Validation of IRGPI

The accuracy of the IRGPI was evaluated in the training cohort, independent validation cohorts and meta-validation cohort by log-rank and ROC curve analyses. To explore whether the IRGPI was an independent variable, it was combined with other clinicopathologic factors (age, gender, tumor stage, smoking habits and alcohol intake) in multivariate Cox analysis. The prognostic value of IRGPI was further verified by log-rank analysis in different subgroups on the basis of clinical characteristics including age, sex and stage.

### Tumor-infiltrating immune cells

CIBERSORT analysis associated with reference mRNA expression values (LM22) were utilized to appraise the relative infiltrating level of 22 immune cells [31]. R package ‘CIBERSORT’ was utilized to analyze. Samples with p-value < 0.05 were remained for the following analysis. The discrepant abundances of different immune cells between the two risk groups were contrasted through the Wilcoxon rank test.

### Functional enrichment

Gene ontology (GO) enrichment analysis was implemented with the R package ‘cluster-Profiler’ to explore potential biological processes and enrichment pathways of the IRGPI. The thresholds were determined by a false discovery rate adjusted *P*-value (FDR) < 0.05.

### Construction of an IRGPI-based predictive nomogram

Using IRGPI and clinicopathologic factors (age, gender, tumor stage, smoking habits and alcohol intake), a nomogram was employed to evaluate prognostic value at 1-, 2- and 3- year survival and the prognostic value was also verified in the meta-validation cohort. The risk score of each factor was computed, and the total score of all factors was taken as the sum of each factor’s risk score. Ultimately, we generated calibration plots to appraise its predictive performance.

### Calculation of tumor immune dysfunction and exclusion (TIDE) score and small molecule drug analysis

The TIDE score (http://tide.dfci.harvard.edu/, accessed on 27 May 2023) was used to evaluate if there were different ICI treatment responses in patients between high-risk and low-risk groups. The CMAP (Connectivity Map; version 1.1.1.43; https://clue.io) database has comprehensive data on the transcriptome of drug interference therapies. We identified potential effective drugs in treating EC through this tool (accessed on 28 May 2023).

### Cell culture

EC cell line KYSE-150 (TCHu236) was purchased from the Cell Bank of the Chinese Academy of Sciences, Shanghai, China. EC cell line KYSE-450 (GDC0633) was purchased from China Center for Type Culture Collection, Wuhan, China. Normal esophageal epithelial cell line Het-1A (CRL-2692) was acquired from American Type Culture Collection. EC cells lines (KYSE-150 and KYSE-450) were cultured with 1640 medium (RPMI, 11875093, Gibco, USA) supplemented with 10% fetal bovine serum (10099141C, Gibco, USA). Normal esophageal epithelial cells (Het-1A) were treated with bronchial epithelial cell basal medium (CC-3170, Lonza, Switzerland). All cell lines were placed in an incubator which contains 5% CO2 at 37°C.

### Quantitative real-time PCR

As described by our previous research (PMID: 33440166), total RNA was extracted with TRIzol reagent (9109, Takara, Japan). RNA was reverse transcribed to cDNA by the High-Capacity cDNA Reverse Transcription Kit (RR047A-5, Takara, Japan). The transcription levels of *IL13RA2, RORC, IL20, SAA2, FABP6, CHGB* were measured by Real-time PCR, SYBR Green PCR Master Mix (HY-KO511, Takara, Japan). Data were analyzed by normalization to the mRNA level of human *β-actin*. The gene primers were as follows: human *β-actin*: F primer, CCTGGCACCCAGCACAAT, R primer, GGGCCGGACTCGTCATAC; *IL13RA2*: F primer, AAAGTTCAGGA-TATGGATTGCGT, R primer, GAAGTACACCTATGCCAGGTTTC; *RORC*: F primer, AGATACCCTCACCTACACCTTG, R primer, CCGCTCAGGGCTGTATTCAA; *IL20*: F primer, ATGAAAGCCTCTAGTCTTGCCT, R primer, GCCCCGTATCTCAGAAAATCC; *SAA2*: F primer, GCTTCTTTTCGTTCCTTGGCG, R primer, GCCGATGTAATT-GGCTTCTCTCA; *FABP6*: F primer, ACCGGCAAGTTCGAGATGG, R primer, CCTTTTCGATTACATCGCTGGA; *CHGB*: F primer, CAGCCAACGCTGCTTCTCA, R primer, GGTTCCTGTTATCCACTGGCA. Three biological replicates were conducted during our qRT-PCR experiment.

### Statistical analysis

All analyses were performed with R software (version 4.0.2). Log-rank test was utilized to contrast the OS between different risk groups. The Cox regression model was adopted to evaluate the prognostic role of IRGPI. For all analysis, two-sided *p*-value < 0.05 was considered as significant.

## Results

### Construction of IRGPI

**Fig. 1:**
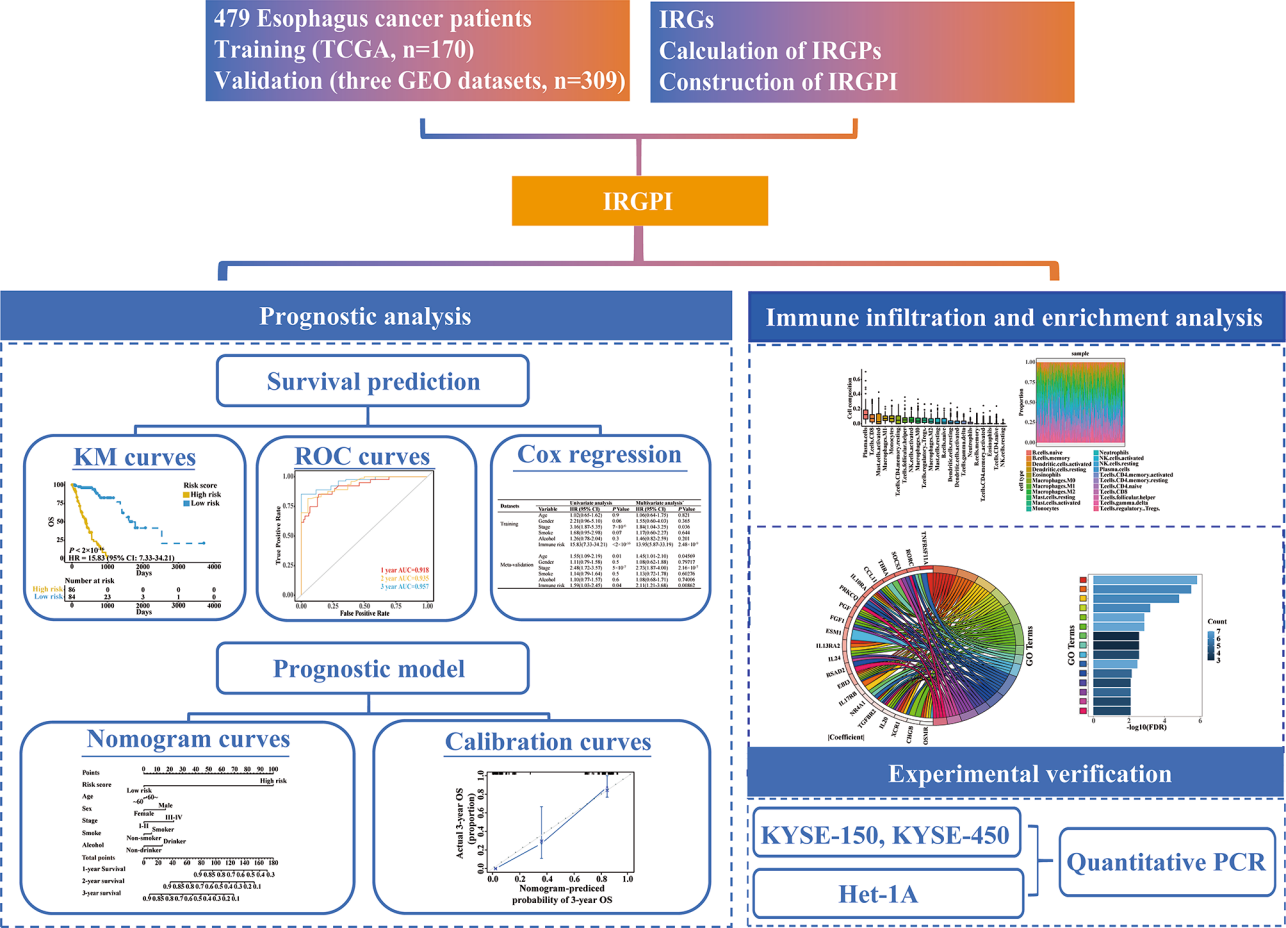
The overview design in this study

In total, 479 EC patients were involved in the study (Table 1). Patient data (170 EC patients) of TCGA dataset were allocated to the training set. Patient data (309 EC patients) from three independent GEO datasets were allocated to the meta-validation cohort (Fig. S1). The overview design in this study was shown in Fig. 1. In total, 694 of the 2483 IRGs measured by all platforms were considered to meet the criteria (MAD > 0.5). Based on these 694 IRGs, 240,471 IRGP were established. After removing 223,503 IRGP with constant ordering and those were not shared in all tested datasets, 16,968 IRGPs were retained as initial candidates for subsequent analysis. The univariate Cox regression model was implemented to accomplish the rough filtration. 912 IRGPs selected by Cox regression model were further filtered out by LASSO analysis. Finally, 21 IRGPs that arose more than 15 times out of 30 LASSO analyses were selected. The 21 IRGPs consisted of 38 unique IRGs (Table S2). The results of multivariate Cox regression were implemented to confirm the IRGP-based prediction and generate IRGPI scores in the training cohort. IRGPI = 1.07740610918684*RSAD2_CRABP1 + 1.80900713722625*CCL11_KLRC1 + 1.0760980156295*HCST_EBI3 + 1.52141607156199*CLDN4_PGF + 0.342479305941611*PPP3CC_ESM1 + 1.26035610621237*ROBO2_ESM1 + 0.455689539588702*TGFBR2_NR4A1 + 0.158188766234162*CTSG_CHGB + 0.714247087056512*MC1R_PROC + 0.327149276848437*XCR1_IL20-1.47563060474962*ITGAL_FGF1-0.0736174398078415*SOCS3_OSMR-0.791568426985749*FABP6_OPRL1-1.3977742637501*NDRG1_CSRP1 + 1.2367977698673*SAA2_CRABP1 + 0.461612771669907*SLPI_IL17RB-2.51795133845012*SOCS3_TNFRSF11A-1.82699508603865*THRA_RORC + 0.230471766652905*HMOX1_ESM1-1.67908325639079*IL10RA_PRKCQ-1.09793933631461*IL24_IL13RA2. The detailed IRGPI construction process was shown in Fig. S2.

**Table 1:** Patients’ demographics and clinicopathologic characteristics in different cohorts

	Training cohort	Validation cohorts		
	TCGA (n = 170)	GSE13898(n = 60)	GSE19417(n = 70)	GSE53625(n = 179)
Age.mean	63.11 ± 11.62	63.17 ± 11.63	-	59.35 ± 9.03
Sex				
Male	144(84.71%)	55(91.67%)	46(65.71%)	146(81.56%)
Female	26(15.29%)	5(8.33%)	24(34.29%)	33(18.44%)
Pathologic stage				
I	18(10.59%)	23(38.33%)	-	10(5.59%)
II	72(42.35%)	16(26.67%)	-	77(43.02%)
III	50(29.41%)	4(6.67%)	-	92(51.40%)
IV	9(5.29%)	4(6.67%)	-	0(0%)
Unknown	21(12.35%)	13(21.67%)	-	0(0%)
Smoke				
Yes	152(89.41%)	43(71.67%)	-	114(63.69%)
No	17(10.00%)	17(28.33%)	-	65(36.31%)
Unknown	1(0.59%)	0(0%)	-	0(0%)
Alcohol				
Yes	117(68.82%)	46(76.67%)	-	106(59.22%)
No	50(29.41%)	14(23.33%)	-	73(40.78%)
Unknown	3(1.76%)	0(0%)	-	0(0%)

### Identification of the prognostic ability of IRGPI

**Fig. 2:**
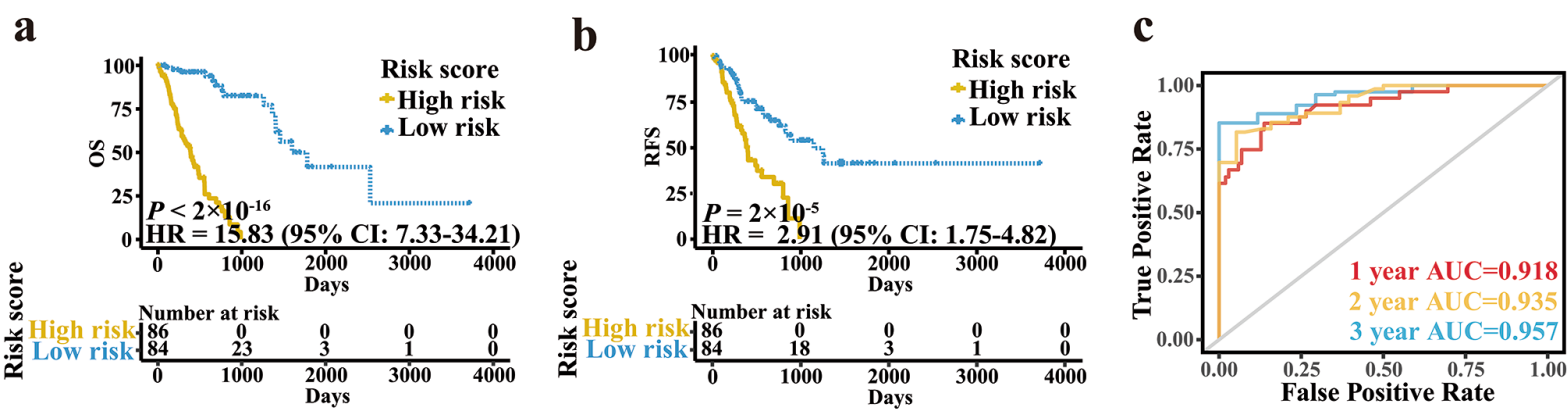
The Kaplan-Meier curves and time-dependent ROC curves in patients of training cohort. (**a**) OS in patients of training dataset classified by the signature. (**b**) RFS in patients of training dataset classified by the signature. (**c**) The time-dependent ROC curves of the IRGPI for 1-, 2- and 3-year OS of patients in training cohort

The ROC curve analysis at 3 years was utilized to choose the optimal cut-off by which EC patients were split into two risk groups. To evaluate the clinical outcomes of the two risk groups, a log-rank test was implemented and the Kaplan-Meier curves indicated that the OS of EC patients in the high-risk group were worse than that in the low-risk group (Fig. 2a, HR 15.83, 95% CI 7.33–34.21; *P* < 2 × 10^− 16^). In addition, the relapse-free survival (RFS) of patients was also calculated and the result was similar to that of OS (Fig. 2b, HR 2.91, 95% CI 1.75–4.82; *P* = 2 × 10^− 5^). To identify the accuracy of the IRGPI in prediction, time-dependent ROC curves were analyzed. The area under the curve (AUC) of predicting 1-,2- and 3-year OS in EC patients was 0.918, 0.935 and 0.957, respectively (Fig. 2c). We also evaluated the predictive ability of IRGPI in various subgroups of patients, which were stratified according to different tumor stage (early stage and late stage), gender (female and male) and age (≤ 60 and > 60). IRGPI also displayed effective predictive value in all these subgroups (Fig. S3). The results of multivariate Cox regression indicated that IRGPI was an independent prognostic factor when age, sex, stage, smoking habits and alcohol intake were adjusted (Table 2, *P* = 2.48×10^− 9^). Similarly, tumor stage was also an independent prognostic factor (Table 2). We further accessed the prognostic ability of the combination of IRGPI and tumor stage. As a result, patients with high IRGPI scores and early stages had the highest OS among all patients (Fig. S4a).

**Table 2:** Identifying the independent prognostic factors through univariate and multivariate Cox analyses

Cohorts		Univariate analysis		Multivariate analysis	
	Variable	HR (95% CI)	*P* Value	HR (95% CI)	*P* Value
Training	Age	1.02(0.65–1.62)	0.9	1.06(0.64–1.75)	0.821
	Gender	2.21(0.96–5.10)	0.06	1.55(0.60–4.03)	0.365
	Stage	3.16(1.87–5.35)	7 × 10^− 6^	1.84(1.04–3.25)	0.036
	Smoke	1.68(0.95–2.98)	0.07	1.17(0.60–2.27)	0.644
	Alcohol	1.26(0.78–2.04)	0.3	1.46(0.82–2.59)	0.201
	Immune risk	15.83(7.33–34.21)	< 2 × 10^− 16^	13.95(5.87–33.19)	2.48 × 10^− 9^
Meta-validation	Age	1.55(1.09–2.19)	0.01	1.45(1.01–2.10)	0.04569
	Gender	1.11(0.79–1.58)	0.5	1.08(0.62–1.88)	0.79717
	Stage	2.48(1.72–3.57)	5 × 10^− 7^	2.73(1.87-4.00)	2.16 × 10^− 7^
	Smoke	1.14(0.79–1.64)	0.5	1.13(0.72–1.78)	0.60276
	Alcohol	1.10(0.77–1.57)	0.6	1.08(0.68–1.71)	0.74006
	Immune risk	1.59(1.03–2.45)	0.04	2.11(1.21–3.68)	0.00862

### Verification of the prognostic ability of IRGPI

**Fig. 3:**
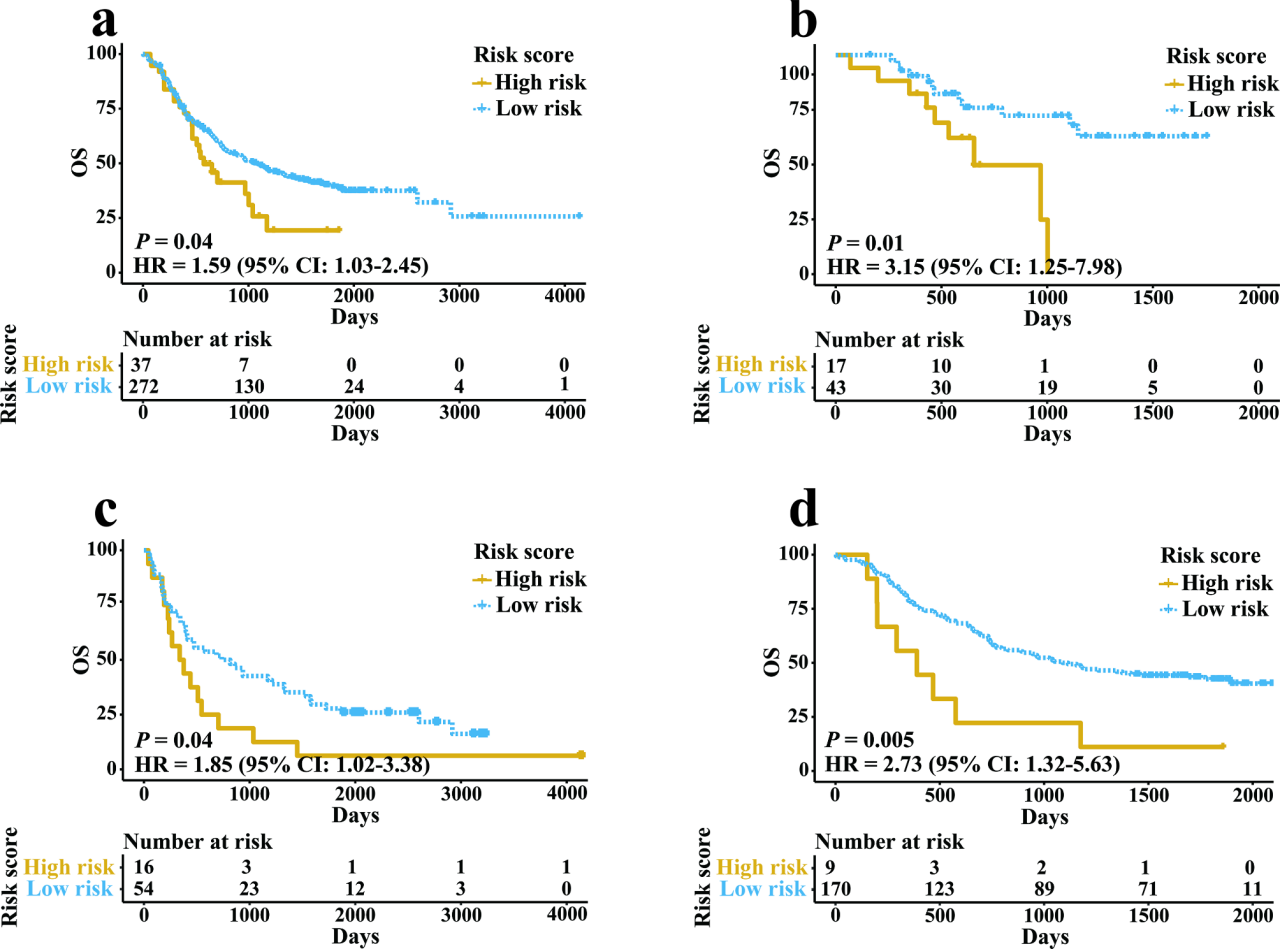
Kaplan-Meier curves in patients of validation sets classified by the signature. (**a**) OS of patients in meta-validation set. (**b**) OS of patients in GSE13898. (**c**) OS of patients in GSE19417. (**d**) OS of patients in GSE53625.

We merged the three independent GEO datasets (GSE13898, GSE19417 and GSE53625) to a meta-validation cohort. Patients in the meta-validation set and each independent validation set were assigned to high- and low-risk groups on the basis of the result of ROC curves. The results of log-rank analysis were consistent with the findings from the training datasets. The OS in EC patients of the high-risk group were worse than that of the low-risk group in meta-validation cohort (Fig. 3a, HR 1.59, 95% CI 1.03–2.45; *P* = 0.04), GSE13898 cohort (Fig. 3b, HR 3.15, 95% CI 1.25–7.98; *P* = 0.01), GSE19417 cohort (Fig. 3c, HR 1.85, 95% CI 1.02–3.38; *P* = 0.04) and GSE53625 cohort (Fig. 3d, HR 2.73, 95% CI 1.32–5.63; *P* = 0.005). In GSE13898 cohort, IRGPI could predict not only OS but also RFS (Fig. S4b). In the meta-validation dataset, when stratifying patients by sex, age and tumor stage, the IRGPI prognostic value remained high for patients ≤ 60 years old, patients with late-stage and male patients (Fig. S5). IRGPI could independently predict the OS of EC patients in the meta-validation cohort after adjusting several clinicopathologic factors (Table 2, HR 2.11, 95% CI 1.21–3.68; *P* = 0.00862). Patients in the low-risk group with early-stage cancer have the best OS in the meta-validation dataset (Fig. S4c).

### Functional enrichment analysis and immune infiltration

**Fig. 4:**
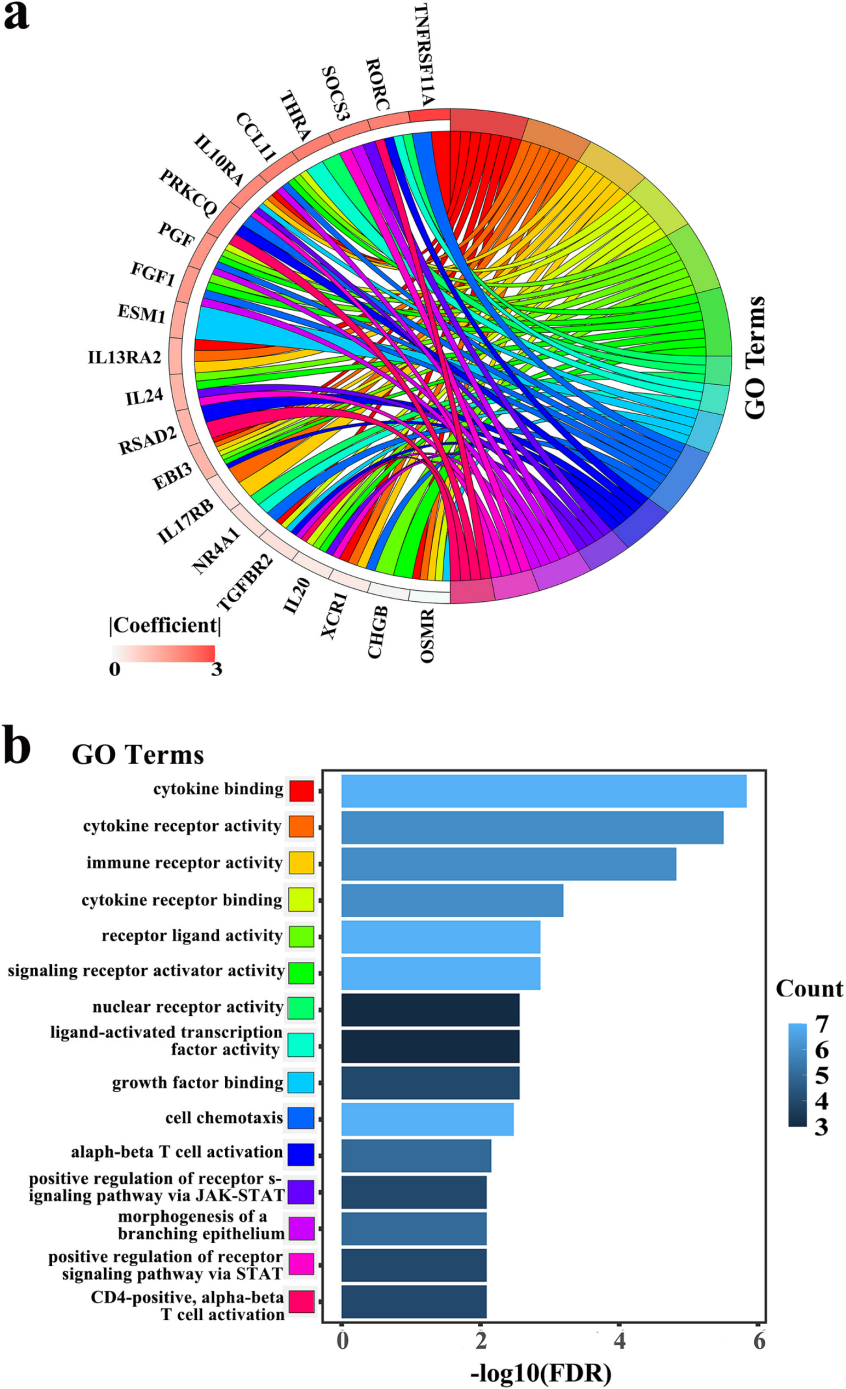
GO enrichment analysis on IRGs of the signature. The GO terms which were the top 15 ranked by FDR were listed. (**a**) The GO terms and their genes. (**b**) FDR and count of each GO term

**Fig. 5:**
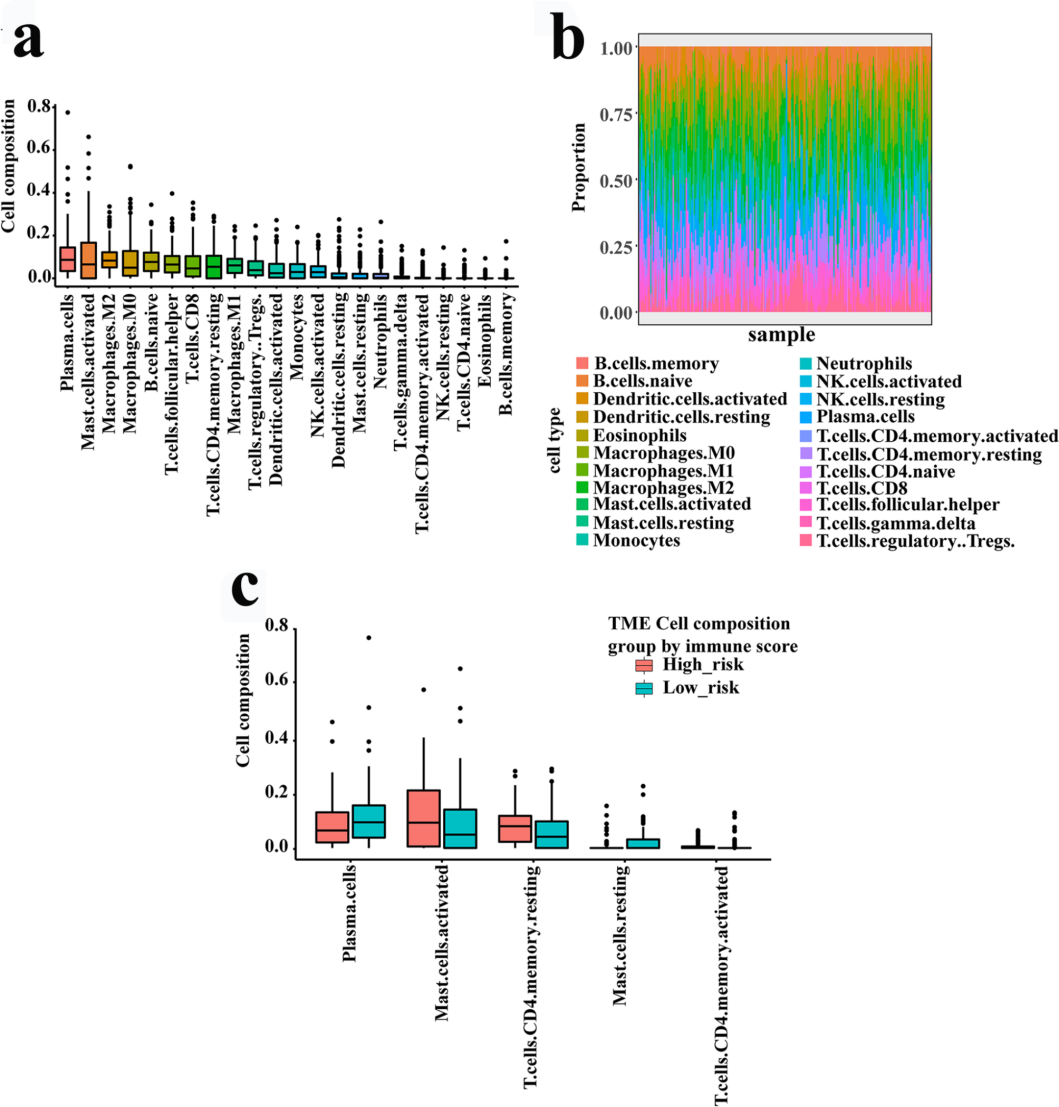
Infiltrating immune cell of the signature. (**a**) 22 immune cells abundance. (**b**) The percentage in 22 immune cells of different samples. (**c**) The immune cells abundance of different risk groups

To acquire the functional activities of these IRGs, we conducted GO analysis and immune infiltration. GO analysis revealed that IRGs in our signature were mainly contained in the chemotaxis, immune response, immune system process, defense response, inflammatory response and cytokine activity (Fig. 4, Table S3). ‘CIBERSORT’ was applied to calculate the abundances of 22 type immune cells in two risk groups. As a result, the number of plasma cells, activated CD4 memory T cells and resting mast cells were significantly higher in the low-risk group. Moreover, the number of activated mast cells and resting CD4 memory T cells were higher in the high-risk group (Fig. 5).

### Integrated prognostic model based on IRGPI and clinicopathologic factors

**Fig. 6:**
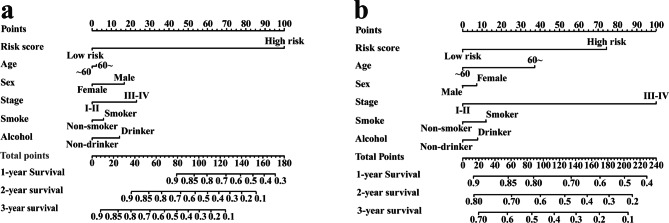
The Nomogram of OS. (**a**) training cohort. (**b**) meta-validation cohort

To obtain the IRGPI based prognostic model for 1-, 2- and 3-year OS rates, we built a nomogram model that incorporated IRGPI and clinicopathologic factors [(age ≤ 60 or > 60), sex, tumor stage, smoking habits and alcohol intake] (Fig. 6a and 6b). The calibration plots were utilized to evaluate the predictive accuracy of the nomogram. The calibration plots revealed good prognostic prediction performance of nomogram in both training dataset and meta-validation dataset (Fig. S6).

### The analysis of the TIDE score and small molecule drug inference

**Fig. 7:**
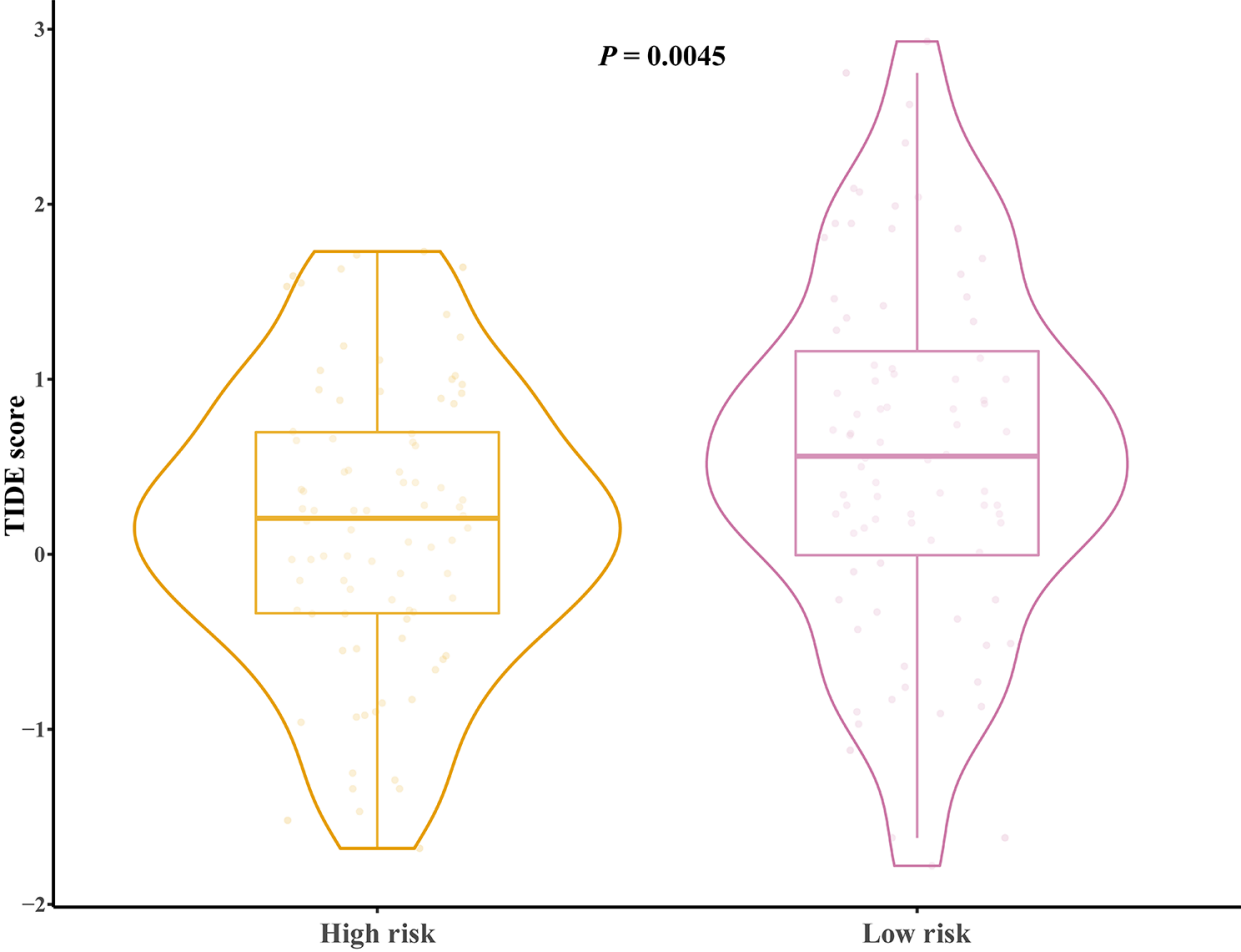
The Correlation analysis between the signature and TIDE score

The TIDE score of different risk groups were calculated and high-risk groups showed a significantly lower TIDE score (*P* = 0.0045, Fig. 7), which suggested patients in high-risk groups have better immunotherapy responses. Moreover, we further identified five potential effective drugs in treating EC through the CMap and they were serotonin receptor agonist, vitamin D receptor agonist, CDC inhibitor, PDGFR receptor inhibitor and leucine rich repeat kinase inhibitor (Table 3).

**Table 3:** The list of compounds which could be as potential drugs in treating EC.

Rank	Score	Name	Description	Target
8549	-87.34	quipazine	Serotonin receptor agonist	HTR2A, HTR3A, HTR3B, HTR1A, HTR1D, HTR2B, HTR2C, HTR6, SLC6A4
8548	-87.05	calcifediol	Vitamin D receptor agonist	VDR
8544	-85.69	KU-C103428N	CDC inhibitor	NFE2
8540	-83.55	linifanib	PDGFR receptor inhibitor	CSF1R, KDR, PDGFRB, FLT1, FLT3, FLT4, CSF1, KIT, PDGFRA, RET, TEK
8530	-76.3	XMD-1150	Leucine rich repeat kinase inhibitor	LRRK2

### Transcription levels of genes involved in IRGPI

**Fig. 8:**
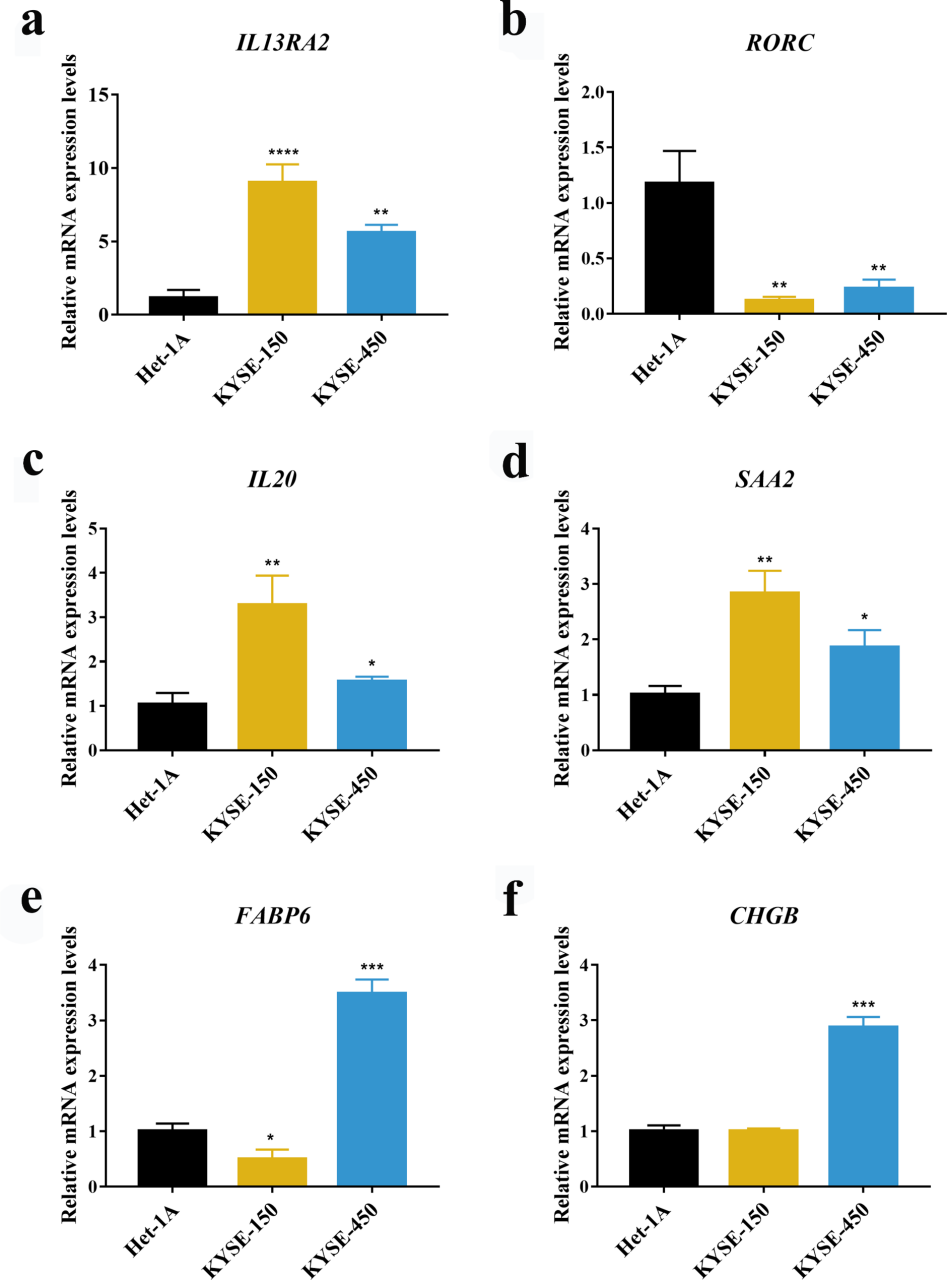
Validation of mRNA expression of genes generated from genes pairs. (**a-f**) Real-time PCR analysis of selected six genes in Het-1 A, KYSE-150 and KYSE-450 cells. Data were presented as the mean ± SE.

To further ascertain the reliability of this IRGPs signature, we selected six genes (*IL13RA2, RORC, IL20, SAA2, FABP6* and *CHGB*) to conduct qRT-PCR experiments in Het-1A cells and EC cells lines (KYSE-150 and KYSE-450) based on the fold change, p-values between tumor and normal tissues gene expression in public databases, as well as high-frequency repeat among gene pairs (Table S4). The mRNA expressions of the majority of genes, including *IL13RA2*, *IL20* and *SAA2*, were persistently higher in tumor cells lines than Het-1A cells (Fig. 8a, 8c and 8d). *RORC* was persistently lower in EC cells lines than Het-1A cells (Fig. 8b). Transcription level of *FABP6* was increased in the KYSE-450 cells but not in the KYSE-150 cells in relative to the Het-1A cells (Fig. 8e). The transcription level of *CHGB* was only upregulated in the KYSE-450 cells (Fig. 8f).

## Discussion

EC, a lethal malignancy, become the sixth leading reason of tumor-related death [2]. Despite the improvement in therapies, the clinical outcomes of EC patients remain poor [4, 5]. Accumulating evidence has verified an association between prognosis and the tumor immune microenvironment. Immunotherapy has been administrated to combat various types of tumors, including EC; however, the cost of immunotherapy associated with EC is prohibitive and patient outcome has been mixed. As such, it’s essential to recognize credible biomarkers to predict EC patients with high risk of mortality or who might be sensitive to immunotherapy. Many researchers have concentrated on the relevance between IRGs expression and tumorigenesis and progression. The prognostic abilities of IRGs were extensively studied [32–34]. Several researches have proposed IRGs-based signatures as prognostic predictors of EC [17–21]. However, the reliability of these signatures is limited. Specifically, one common drawback of these signatures is the lack of accurate normalization to reduce the biological heterogeneity when processing samples measured by different platforms. IRGPs signatures are on basis of the relative gene expression ranking of paired genes in each patient and eliminates the bias of normalization. IRGPs-based signatures have been proven to be effective prognostic biomarkers in many solid tumors, including gastric tumor, lung tumor, colorectal tumor, and renal cell cancer [27–30]. Based on the TCGA, GEO and ImmPort datasets, we constructed an IRGPs signature using 21 IRGPs. EC patients were segmented into two risk groups depending on the IRGPI results above, with survival analyses revealing a significant difference between different groups. When patients were further stratified into different subgroups according to age, sex and tumor stage, differences remained regardless of these clinicopathologic features. The nomogram based on IRGPI and clinicopathologic features including age, sex and tumor stages could quantitatively predict the OS of patients with EC.

When it comes to the researches identifying cancer prognostic biomarkers, all strives aim to obtain robust results. To achieve this goal, we had done the following four things. Firstly, we validated the expression levels of six selected genes from IRGPI in KYSE-150 and KYSE-450. Our research is a combination of bioinformatics and experimentation. Based on these results and related literatures which showed that these genes were related to tumorigenesis [35–40], we infer that they are associated with esophageal tumorigenesis. After searching literatures in PubMed with “immune-related gene pair [Title/Abstract]” as keywords, we found 25 articles and none had their own experiments validation. Secondly, researches with multiple platforms could obtain relatively robust results [41]. This study included adequate external validation datasets with three GEO datasets obtained from multiple platforms, as shown in Fig. 3. Moreover, its abilities in distinguishing different OS in patients with different pathological stages, age and sex groups were verified in both the training cohort and validation cohorts, as shown in Fig. S3 and Fig. S5. Thirdly, the signature was developed based on a robust calculation process. At first, the association between each IRGP and patients’ OS in the training dataset was assessed by the univariate Cox regression model. After rough filtration, the LASSO was further carried out to minimize overfitting. To improve the robustness of selected IRGPs, we randomly divided the training set into new training sets and test sets through a ratio of 2:1, and then duplicated this procedure 30 times. The LASSO was then used in the 30 training sets to choose those IRGPs with a frequency > 15. Then, we adopted the multivariate Cox regression analysis to construct the IRGP-based model, which consisted of relevant IRGPI and respective coefficients. Similarly, previous studies have utilized advanced machine learning methods to obtain more robust and accurate results [42–46]. Zhao et al. investigated cardiotoxicity related with hERG channel blockers using molecular fingerprints and graph attention mechanism [42]. Hu et al. conducted the association analysis between gene function and cell surface protein based on single-cell multi-omics data [43]. A study used a deep learning method to predict metabolite-disease associations via a graph neural network [44]. There were also some researches which focused on predicting lncRNA-miRNA interactions based on graph convolution network with conditional random field and network distance analysis [45, 46]. These interesting works would provide valuable directions for us. Fourthly, biological functions, immune infiltration and bioinformatics analysis were combined to confirm the reliability of our results. Pathway enrichment analysis showed that our signature-related genes were mainly involved in immune and inflammatory response, cytokine activity and chemotaxis. A significantly higher infiltration level of plasma cells and activated CD4 memory T cells was found in the low-risk group versus the high-risk group. Those results could provide clues for our future experimental researches.

GO analysis was conducted to obtain the functional activities of those IRGs consisting of IRGPs. The pathways associated with IRGPs mainly contained in cytokine, chemotaxis, T cell activation, receptor and ligand activation, morphogenesis of epithelium and angiogenesis (Fig. 4). Namely, most are involved in specific immune activities, and a few are receptor and ligand activation, and tumorigenesis. The results indicated that IRGs included in IRGPI participated in some specific immune and tumorigenesis activities. The results of immune cell abundance analysis revealed that the number of activated CD4 memory T cells, plasma cells and resting mast cells were significantly higher in the low-risk EC patients. Besides, resting CD4 memory T cells and activated mast cells have an inverse result. In recent years, evaluation of immune activity in multiple cancers has revealed that B cells and plasma cells may act as biomarkers to predict the effectiveness of immunotherapy. Plasma cells in tumor microenvironment are related to ameliorative outcomes [31]. Yao et al. also found an association between high tumor‑infiltrating plasma cells and favorable OS prognosis in EC patients [31]. The results of our study were similar to these previous studies, whereby we found the abundance of activated CD4 memory T cells were lower in the high-risk group. Indeed, activated CD4 memory T cells was related to favorable outcome in several tumors [27, 33]. To a certain extent, the differences in immune cells composition in two groups may explain the prognostic value of the IRGPI in EC.

To identify the reliability of the IRGPs signature in EC, we conducted qRT-PCR to investigate the transcription levels of six selected genes. The *IL13RA2*, *SAA2* and *IL20* have a significantly higher expression level, whereas the transcriptional level of RORC was lower, in EC cells lines than that in Het-1A cells. *IL13RA2* participated in the regulation of *IL13* and *IL4* [32]. *IL13RA2* is overexpressed in various tumors, such as glioblastoma, pancreatic tumor, ovarian tumor and head and neck tumor [35, 36, 47, 48]. Thus, we surmise that *IL13RA2* possesses an essential role in the tumorigenesis of EC, and further work is needed to investigate the mechanism of *IL13RA2* in EC. Human *SAA1* and *SAA2* encode identical proteins. The *SAA2* protein belongs to the SAA protein [49] and participates in the progression of several tumors. It is reported that *SAA2* is significantly increased when renal cell carcinoma (RCC) patients have the highest Fuhrman grade [50]. *SAA2* could also be a potential predictor of OS in RCC [50]. Indeed, *SAA2* was significantly increased in lung cancer and had effective diagnostic value in lung, endometrial and colorectal tumors [37, 38, 51]. Wang et al. showed that *SAA* levels were related with poor survival [41, 52]. According to our results and previous studies, it is a reasonable inference that *SAA2* is related to tumor tumorigenesis and poor survival of EC patients. However, this assumption needs further study. RORC, a member of ROR subfamily, has been studied in various cancer cells and their corresponding tumor microenvironment in recent years, with results showing that it might possess effective prognostic value in both lung and breast cancers. RORC could also be as a promising molecular target for the therapy of prostate tumor [39]. The results of our research indicated that RORC was downregulated in EC cells when compared with Het-1A cells. *IL20* is a part of *IL10* family and plays important roles in various immunopathological diseases [53]. Previous studies have indicated that *IL20* takes part in cancer progression in several tumors [54–57]. However, researches of *IL2*0 in EC were limited. Increased expression of *IL20* was found in EC of previous microarray analysis [40], but the potential mechanisms of *IL20* in EC is unknow yet. Our in vitro experiment showed that *IL20* has a significantly higher expression in EC cells than in Het-1A cells. This result is in accordance with the increased expression of *IL20* in several cancers including EC. Further study is warranted to explore the role of IL20 on EC tumorigenesis.

It should be noted that there are some limitations in our research. First, despite four unique datasets being included, only 479 samples were analyzed and more samples are needed for further validation. Second, considering the results of this study were obtained from retrospective analyses, prospective study is required to confirm the efficiency of this IRGPs signature model.

## Conclusion

In conclusion, The IRGPI, constructed in this study, can both stratified EC into different risk groups and predict the prognosis of EC, effectively. The results of our study provide promising perspectives for selecting patients who are sensitive to immunotherapy and pave the way for EC treatment.

## Electronic supplementary material

Below is the link to the electronic supplementary material.

**Fig. S1**. Overview of the construction and validation of immune-related gene pairs signature (IRGPI). Four datasets were included in this study. TCGA dataset was used for training cohort, and GSE13898, GSE19417, GSE53625 were merged to the meta-validation cohort. The training cohort was used to build an IRGPI. The IRGPI was verified on the meta-validation cohort and independent validation cohorts.

**Fig. S2**. Flow chart of IRGPI construction.

**Fig. S3**. Kaplan-Meier curves in different subgroups’ cases of training set. (a) OS of cases with early stage. (b) OS of male cases. (c) OS of cases ≤ 60 years old. (d) OS of cases with late stage. (e) OS of female cases. (f) OS of cases > 60 years old

**Fig. S4**. The Kaplan-Meier curves. (a) OS among patients in training cohort stratified by IRGPI and tumor stage. (b) Relapse-free survival among patients in validation cohort of GSE13898. (c) OS among patients in meta-validation cohort stratified by IRGPI and tumor stage.

**Table S1**. Details about datasets used in this study.

**Table S3**. The significant biological processes enriched by genes consisted in the IRGPI.

**Table S2**. Model information about the IRGPI

**Table. S4**. The expression levels of six genes in training and validation dataset.

**Fig. S5**. Kaplan-Meier curves in different subgroups’ cases of meta-validation dataset. (a) OS of cases in early stage. (b) OS of male cases. (c) OS of cases ≤ 60 years old. (d) OS of cases in late stage. (e) OS of female cases. (f) OS of cases > 60 years old.

**Fig. S6**. Nomogram evaluation for predicting 1-, 2- and 3-year OS. (a-c) Calibration plots of the nomogram for predicting the probability of OS at 1 (a), 2 (b) and 3 years (c) in training cohort. (df) Calibration plots of the nomogram for predicting the probability of OS at 1 (d), 2 (e), 3 years (f) in meta-validation cohort.

## Data Availability

The data of bioinformatics analysis is available at TCGA and GEO database, data of experimental research will be provided upon request. Cancer Genome Atlas (TCGA): https://xenabrowser.net/datapages/; GSE13898: https://www.ncbi.nlm.nih.gov/geo/query/acc.cgi?acc=GSE13898; GSE19417: https://www.ncbi.nlm.nih.gov/geo/query/acc.cgi?acc=GSE19417; GSE52625: https://www.ncbi.nlm.nih.gov/geo/query/acc.cgi?acc=GSE52625. Further information and requests for resources and reagents should be directed to and will be fulfilled by Biao Xie, kybiao@cqmu.edu.cn.
