# Supplementary material for: A robust immune-related gene pairs signature for predicting the overall survival of esophageal cancer
Source: BMC Genomics. 2023 Jul 10;24:385. doi: 10.1186/s12864-023-09496-x (PMC10332031; doi:10.1186/s12864-023-09496-x)
Supplement: Supplementary file 1 — Fig. S1. Overview of the construction and validation of immune-related gene pairs signature (IRGPI). Four datasets were included in this study. TCGA dataset was used for training cohort, and GSE13898, GSE19417, GSE53625 were merged to the meta-validation cohort. The training cohort was used to build an IRGPI. The IRGPI was verified on the meta-validation cohort and independent validation cohorts. [file 12864_2023_9496_MOESM1_ESM.pdf]

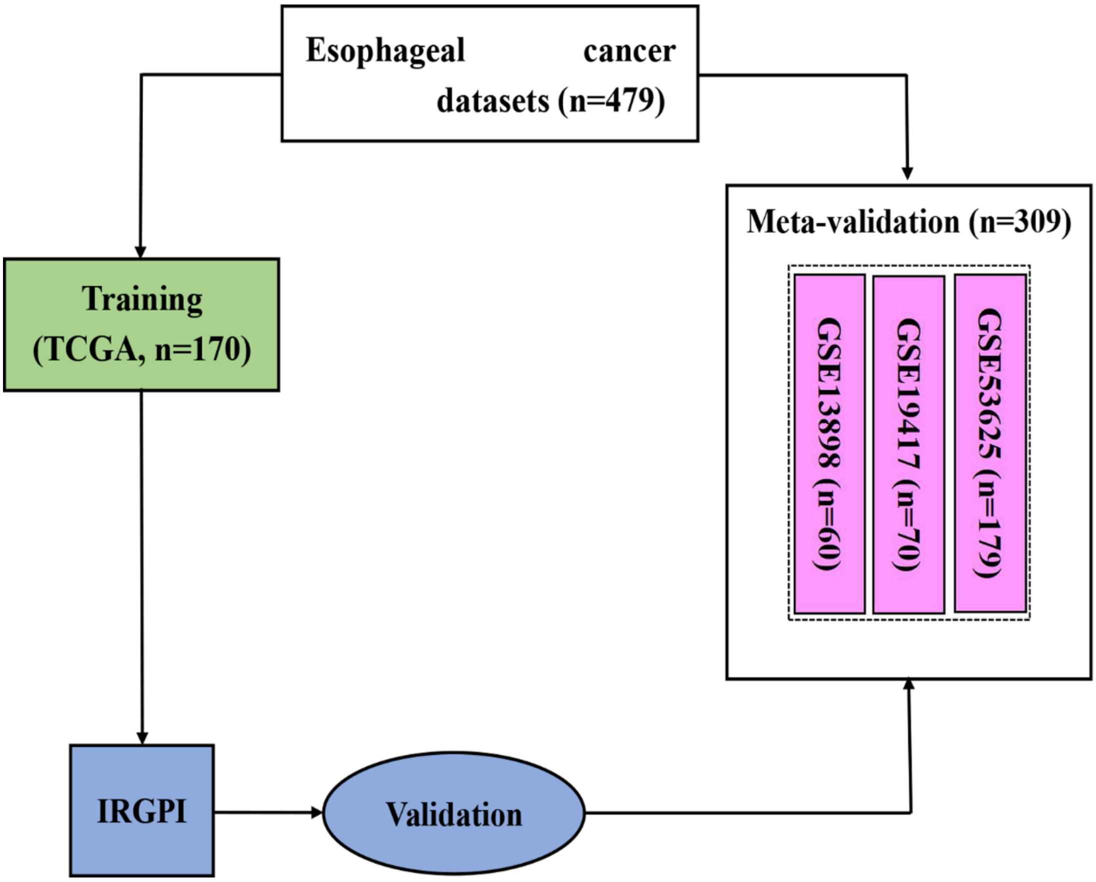

Fig. S1. Overview of the construction and validation of immune-related gene pairs signature (IRGPI). Four datasets were included in this study. TCGA dataset was used for training cohort, and GSE13898, GSE19417, GSE53625 were merged to the meta-validation cohort. The training cohort was used to build an IRGPI. The IRGPI was verified on the meta-validation cohort and independent validation cohorts.
