# Supplementary figures and images for: A robust immune-related gene pairs signature for predicting the overall survival of esophageal cancer
Source: BMC Genomics. 2023 Jul 10;24:385. doi: 10.1186/s12864-023-09496-x (PMC10332031; doi:10.1186/s12864-023-09496-x)

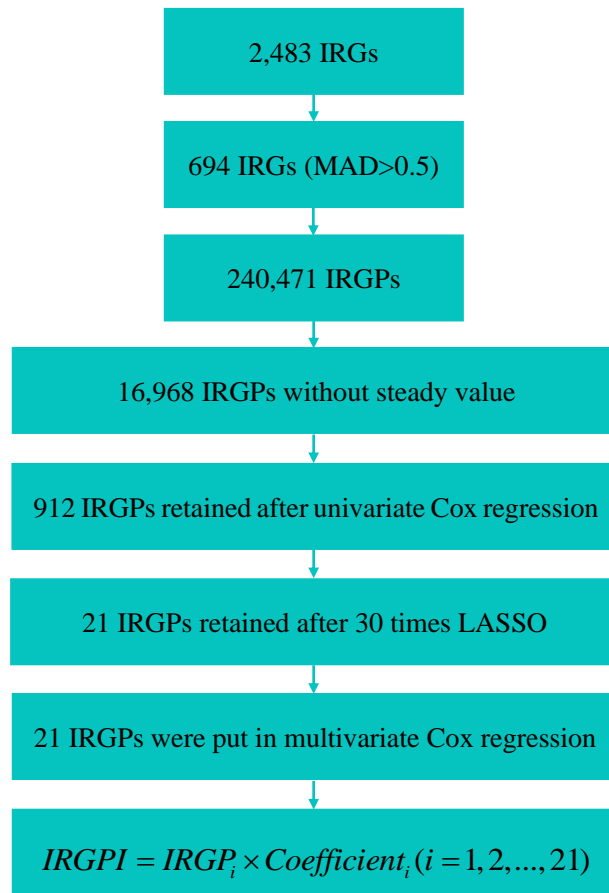

**Fig. S2.** Flow chart of IRGPI construction.

Supplement: Supplementary file 2 — Fig. S2. Flow chart of IRGPI construction. [file 12864_2023_9496_MOESM2_ESM.pdf]
