# Supplementary material for: A robust immune-related gene pairs signature for predicting the overall survival of esophageal cancer
Source: BMC Genomics. 2023 Jul 10;24:385. doi: 10.1186/s12864-023-09496-x (PMC10332031; doi:10.1186/s12864-023-09496-x)
Supplement: Supplementary file 4 — Fig. S4. The Kaplan-Meier curves. (a) OS among patients in training cohort stratified by IRGPI and tumor stage. (b) Relapse-free survival among patients in validation cohort of GSE13898. (c) OS among patients in meta-validation cohort stratified by IRGPI and tumor stage. [file 12864_2023_9496_MOESM4_ESM.pdf]

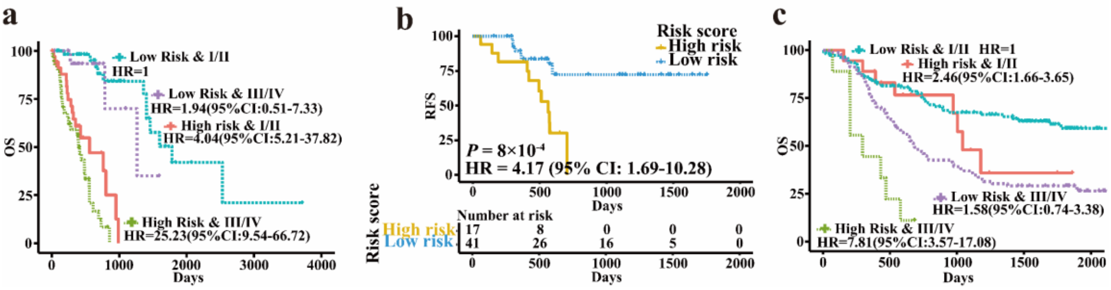

Fig. S4. The Kaplan-Meier curves. (a) OS among patients in training cohort stratified by IRGPI and tumor stage. (b) Relapse-free survival among patients in validation cohort of GSE13898. (c) OS among patients in meta-validation cohort stratified by IRGPI and tumor stage.
