# Supplementary material for: A robust immune-related gene pairs signature for predicting the overall survival of esophageal cancer
Source: BMC Genomics. 2023 Jul 10;24:385. doi: 10.1186/s12864-023-09496-x (PMC10332031; doi:10.1186/s12864-023-09496-x)
Supplement: Supplementary file 5 — Table S1. Details about datasets used in this study. [file 12864_2023_9496_MOESM5_ESM.pdf]

Table S1. Details about datasets used in this study.

Accession number	Platform	Samples
TCGA	Illumina HiSeq	170
GSE13898	Illumina human-6 v2.0 expression beadchip	60
GSE19417	Rosetta/Merck Human 44k 1.1 microarray	70
GSE52625	Agilent-038314 CBC Homo sapiens mRNA microarray V2.0	179
