# Supplementary material for: A robust immune-related gene pairs signature for predicting the overall survival of esophageal cancer
Source: BMC Genomics. 2023 Jul 10;24:385. doi: 10.1186/s12864-023-09496-x (PMC10332031; doi:10.1186/s12864-023-09496-x)
Supplement: Supplementary file 6 — Table S3. The significant biological processes enriched by genes consisted in the IRGPI. [file 12864_2023_9496_MOESM6_ESM.pdf]

Table S3. The significant biological processes enriched by genes consisted in the IRGPI.

GO ID	Category	Description	FDR	Count
GO:0060326	BP	cell chemotaxis	0.003317354	7
GO:0046631	BP	alpha-beta T cell activation	0.006977681	5
GO:0046427	BP	positive regulation of receptor signaling pathway via JAK-STAT	0.008115008	4
GO:0061138	BP	morphogenesis of a branching epithelium	0.008115	5
GO:1904894	BP	positive regulation of receptor signaling pathway via STAT	0.008115	4
GO:0035710	BP	CD4-positive, alpha-beta T cell activation	0.008115	4
GO:0002040	BP	sprouting angiogenesis	0.008115	5
GO:0001763	BP	morphogenesis of a branching structure	0.008115	5
GO:0045766	BP	positive regulation of angiogenesis	0.008175	5
GO:0050679	BP	positive regulation of epithelial cell proliferation	0.008175	5
GO:0001938	BP	positive regulation of endothelial cell proliferation	0.009222	4
GO:1904018	BP	positive regulation of vasculature development	0.011329	5
GO:0002887	BP	negative regulation of myeloid leukocyte mediated immunity	0.015593	2
GO:0046425	BP	regulation of receptor signaling pathway via JAK-STAT	0.015593	4
GO:0050727	BP	regulation of inflammatory response	0.015593	6
GO:1904892	BP	regulation of receptor signaling pathway via STAT	0.015593	4
GO:0048732	BP	gland development	0.015593	6
GO:0045576	BP	mast cell activation	0.015593	3
GO:0002551	BP	mast cell chemotaxis	0.015593	2
GO:0033004	BP	negative regulation of mast cell activation	0.015593	2
GO:0043301	BP	negative regulation of leukocyte degranulation	0.015593	2
GO:0048754	BP	branching morphogenesis of an epithelial tube	0.015593	4
GO:0046635	BP	positive regulation of alpha-beta T cell activation	0.015783	3
GO:0048645	BP	animal organ formation	0.016019	3
GO:0097531	BP	mast cell migration	0.016019	2
GO:0007259	BP	receptor signaling pathway via JAK-STAT	0.016358	4
GO:0048511	BP	rhythmic process	0.016907	5
GO:0042110	BP	T cell activation	0.017688	6
GO:0042531	BP	positive regulation of tyrosine phosphorylation of STAT protein	0.017746	3
GO:0097696	BP	receptor signaling pathway via STAT	0.017746	4
GO:0031100	BP	animal organ regeneration	0.019583	3
GO:0001936	BP	regulation of endothelial cell proliferation	0.020545	4
GO:0032496	BP	response to lipopolysaccharide	0.020942	5
GO:0110110	BP	positive regulation of animal organ	0.02493	3

		morphogenesis		
GO:0001935	BP	endothelial cell proliferation	0.02493	4
GO:0042509	BP	regulation of tyrosine phosphorylation of STAT protein	0.02493	3
GO:0002237	BP	response to molecule of bacterial origin	0.02493	5
GO:0007260	BP	tyrosine phosphorylation of STAT protein	0.026351	3
GO:0046641	BP	positive regulation of alpha-beta T cell proliferation	0.027367	2
GO:0002460	BP	adaptive immune response based on somatic recombination of immune receptors built from immunoglobulin superfamily domains	0.027429	5
GO:0007623	BP	circadian rhythm	0.029113	4
GO:0046634	BP	regulation of alpha-beta T cell activation	0.029113	3
GO:0001657	BP	ureteric bud development	0.029113	3
GO:0072163	BP	mesonephric epithelium development	0.029113	3
GO:0072164	BP	mesonephric tubule development	0.029113	3
GO:0001759	BP	organ induction	0.029113	2
GO:0003272	BP	endocardial cushion formation	0.029113	2
GO:1903306	BP	negative regulation of regulated secretory pathway	0.029113	2
GO:0042102	BP	positive regulation of T cell proliferation	0.029389	3
GO:0050678	BP	regulation of epithelial cell proliferation	0.029389	5
GO:0001823	BP	mesonephros development	0.029988	3
GO:0045765	BP	regulation of angiogenesis	0.030892	5
GO:0046632	BP	alpha-beta T cell differentiation	0.031348	3
GO:0003148	BP	outflow tract septum morphogenesis	0.032785	2
GO:0002526	BP	acute inflammatory response	0.034493	3
GO:0030099	BP	myeloid cell differentiation	0.038591	5
GO:0002675	BP	positive regulation of acute inflammatory response	0.040045	2
GO:0072538	BP	T-helper 17 type immune response	0.040045	2
GO:1901342	BP	regulation of vasculature development	0.040045	5
GO:0035767	BP	endothelial cell chemotaxis	0.040045	2
GO:0043304	BP	regulation of mast cell degranulation	0.040045	2
GO:0022612	BP	gland morphogenesis	0.040045	3
GO:0033006	BP	regulation of mast cell activation involved in immune response	0.040045	2
GO:0045920	BP	negative regulation of exocytosis	0.040045	2
GO:0046627	BP	negative regulation of insulin receptor signaling pathway	0.040045	2
GO:0046640	BP	regulation of alpha-beta T cell proliferation	0.040045	2
GO:0050673	BP	epithelial cell proliferation	0.04011	5
GO:0003203	BP	endocardial cushion morphogenesis	0.040799	2
GO:0036314	BP	response to sterol	0.040799	2

GO:0070670	BP	response to interleukin-4	0.040799	2
GO:1900077	BP	negative regulation of cellular response to insulin stimulus	0.040799	2
GO:0098742	BP	cell-cell adhesion via plasma-membrane adhesion molecules	0.040799	4
GO:0002701	BP	negative regulation of production of molecular mediator of immune response	0.040799	2
GO:0010092	BP	specification of animal organ identity	0.040799	2
GO:0031128	BP	developmental induction	0.040799	2
GO:0046633	BP	alpha-beta T cell proliferation	0.040799	2
GO:2000516	BP	positive regulation of CD4-positive, alpha-beta T cell activation	0.040799	2
GO:0050671	BP	positive regulation of lymphocyte proliferation	0.043432	3
GO:0032946	BP	positive regulation of mononuclear cell proliferation	0.043709	3
GO:0003156	BP	regulation of animal organ formation	0.043709	2
GO:0030879	BP	mammary gland development	0.046681	3
GO:0072073	BP	kidney epithelium development	0.046681	3
GO:0002700	BP	regulation of production of molecular mediator of immune response	0.047977	3
GO:2000826	BP	regulation of heart morphogenesis	0.048371	2
GO:0009897	CC	external side of plasma membrane	0.011787	6
GO:0019955	MF	cytokine binding	1.46E-06	7
GO:0004896	MF	cytokine receptor activity	3.14E-06	6
GO:0140375	MF	immune receptor activity	1.50E-05	6
GO:0005126	MF	cytokine receptor binding	0.000637	6
GO:0048018	MF	receptor ligand activity	0.001366	7
GO:0030546	MF	signaling receptor activator activity	0.001366	7
GO:0004879	MF	nuclear receptor activity	0.002725	3
GO:0098531	MF	ligand-activated transcription factor activity	0.002725	3
GO:0019838	MF	growth factor binding	0.002725	4
GO:0005539	MF	glycosaminoglycan binding	0.017495	4
GO:0005125	MF	cytokine activity	0.017495	4
GO:0070851	MF	growth factor receptor binding	0.035891	3
GO:0008528	MF	G protein-coupled peptide receptor activity	0.037973	3
GO:0001653	MF	peptide receptor activity	0.038002	3
GO:0008201	MF	heparin binding	0.047683	3
