# Supplementary material for: A robust immune-related gene pairs signature for predicting the overall survival of esophageal cancer
Source: BMC Genomics. 2023 Jul 10;24:385. doi: 10.1186/s12864-023-09496-x (PMC10332031; doi:10.1186/s12864-023-09496-x)
Supplement: Supplementary file 7 — Table S2. Model information about the IRGPI [file 12864_2023_9496_MOESM7_ESM.pdf]

Table S2. Model information about the IRGPI

IRG 1	Full name	Immune processes	IRG 2	Full name	Immune processes	Coefficient
RSAD2	radical S-adenosyl methionine domain containing 2	Antimicrobials	CRABP1	cellular retinoic acid binding protein 1	Antimicrobials	1.07740610918684
CCL11	C-C motif chemokine ligand 11	Multiple	KLRC1	killer cell lectin like receptor C1	Multiple	1.80900713722625
HCST	hematopoietic cell signal transducer	Natural Killer Cell Cytotoxicity	EBI3	Epstein-Barr virus induced 3	Cytokines	1.0760980156295
CLDN4	claudin 4	Antimicrobials	PGF	placental growth factor	Cytokines	1.52141607156199
PPP3CC	protein phosphatase 3 catalytic subunit gamma	Multiple	ESM1	endothelial cell specific molecule 1	Cytokines	0.342479305941611
ROBO2	roundabout guidance receptor 2	Multiple	ESM1	endothelial cell specific molecule 1	Cytokines	1.26035610621237
TGFBR2	transforming growth factor beta receptor 2	Multiple	NR4A1	nuclear receptor subfamily 4 group A member 1	Cytokine Receptors	0.455689539588702
CTSG	cathepsin G	Multiple	CHGB	chromogranin B	Cytokines	0.158188766234162
MC1R	melanocortin 1 receptor	Cytokine Receptors	PROC	protein C, inactivator of coagulation factors Va and VIIIa	Antimicrobials	0.714247087056512
XCR1	X-C motif chemokine receptor 1	Multiple	IL20	interleukin 20	Multiple	0.327149276848437
ITGAL	integrin subunit alpha L	Natural Killer Cell Cytotoxicity	FGF1	fibroblast growth factor 1	Cytokines	-1.47563060474962
SOCS3	suppressor of cytokine signaling 3	Antimicrobials	OSMR	Oncostatin M receptor	Cytokine Receptors	-0.0736174398078415
FABP6	fatty acid binding protein 6	Antimicrobials	OPRL1	opioid related nociceptin receptor 1	Cytokine Receptors	-0.791568426985749
NDRG1	N-myc downstream regulated 1	Antimicrobials	CSRP1	cysteine and glycine rich protein 1	Antimicrobials	-1.3977742637501
SAA2	serum amyloid A2	Multiple	CRABP1	cellular retinoic acid binding protein 1	Antimicrobials	1.2367977698673
SLPI	secretory leukocyte peptidase inhibitor	Antimicrobials	IL17RB	interleukin 17 receptor B	Multiple	0.461612771669907
SOCS3	suppressor of cytokine signaling 3	Antimicrobials	TNFRSF11A	TNF receptor superfamily member 11a	Multiple	-2.51795133845012
THRA	thyroid hormone receptor alpha	Cytokine_Receptors	RORC	RAR related orphan receptor C	Cytokine Receptors	-1.82699508603865
HMOX1	heme oxygenase 1	Antimicrobials	ESM1	endothelial cell specific molecule 1	Cytokines	0.230471766652905
