# Supplementary material for: A robust immune-related gene pairs signature for predicting the overall survival of esophageal cancer
Source: BMC Genomics. 2023 Jul 10;24:385. doi: 10.1186/s12864-023-09496-x (PMC10332031; doi:10.1186/s12864-023-09496-x)
Supplement: Supplementary file 8 — Table. S4. The expression levels of six genes in training and validation dataset. [file 12864_2023_9496_MOESM8_ESM.pdf]

Table. S4. The expression levels of six genes in training and validation dataset.

Genes	Tumor tissues	Normal tissues	Fold change	*P*-value*
**TCGA**				
IL13RA2	3.71±2.14	1.35±1.36	2.76	0.00039057
IL20	2.12±2.04	0.40±0.51	5.30	0.005825023
SAA2	5.67±2.52	2.13±1.69	2.67	7.82×10^{-6}
FABP6	4.20±2.30	2.01±1.85	2.09	0.0022409
CHGB	3.10±2.42	6.15±2.40	0.50	7.32×10^{-5}
RORC	5.63±2.77	7.99±3.75	0.70	0.007951905
**Meta-validation dataset**				
IL13RA2	7.34±1.71	6.22±1.42	1.18	9.86×10^{-14}
IL20	8.19±1.50	7.77±0.82	1.05	0.0001673
SAA2	8.56±2.08	8.54±2.41	1.01	0.9551
FABP6	10.02±2.33	10.51±1.73	0.95	0.009206
CHGB	8.31±1.94	8.22±1.39	1.01	0.5716
RORC	7.08±1.21	10.23±1.87	0.69	2.20×10^{-16}

**P*-value was derived from t-test.
