# Supplementary material for: A robust immune-related gene pairs signature for predicting the overall survival of esophageal cancer
Source: BMC Genomics. 2023 Jul 10;24:385. doi: 10.1186/s12864-023-09496-x (PMC10332031; doi:10.1186/s12864-023-09496-x)
Supplement: Supplementary file 9 — Fig. S5. Kaplan-Meier curves in different subgroups’ cases of meta-validation dataset. (a) OS of cases in early stage. (b) OS of male cases. (c) OS of cases ≤ 60 years old. (d) OS of cases in late stage. (e) OS of female cases. (f) OS of cases > 60 years old. [file 12864_2023_9496_MOESM9_ESM.pdf]

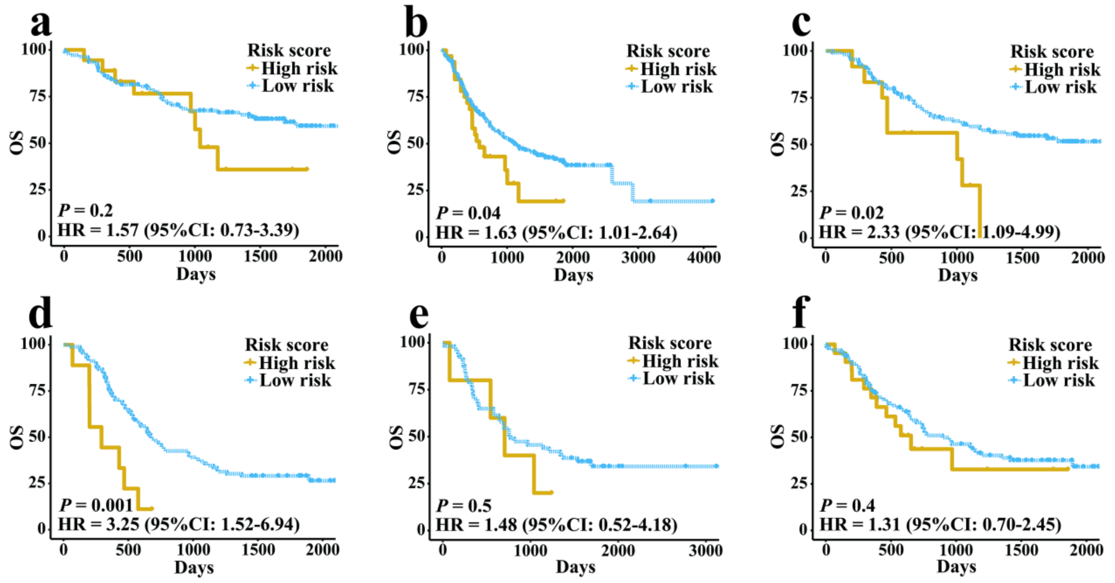

Fig. S5. Kaplan-Meier curves in different subgroups' cases of meta-validation dataset. (**a**) OS of cases in early stage. (**b**) OS of male cases. (**c**) OS of cases ≤ 60 years old. (**d**) OS of cases in late stage. (**e**) OS of female cases. (**f**) OS of cases > 60 years old.
