# Supplementary material for: A robust immune-related gene pairs signature for predicting the overall survival of esophageal cancer
Source: BMC Genomics. 2023 Jul 10;24:385. doi: 10.1186/s12864-023-09496-x (PMC10332031; doi:10.1186/s12864-023-09496-x)
Supplement: Supplementary file 10 — Fig. S6. Nomogram evaluation for predicting 1-, 2- and 3-year OS. (a-c) Calibration plots of the nomogram for predicting the probability of OS at 1 (a), 2 (b) and 3 years (c) in training cohort. (df) Calibration plots of the nomogram for predicting the probability of OS at 1 (d), 2 (e), 3 years (f) in meta-validation cohort. [file 12864_2023_9496_MOESM10_ESM.pdf]

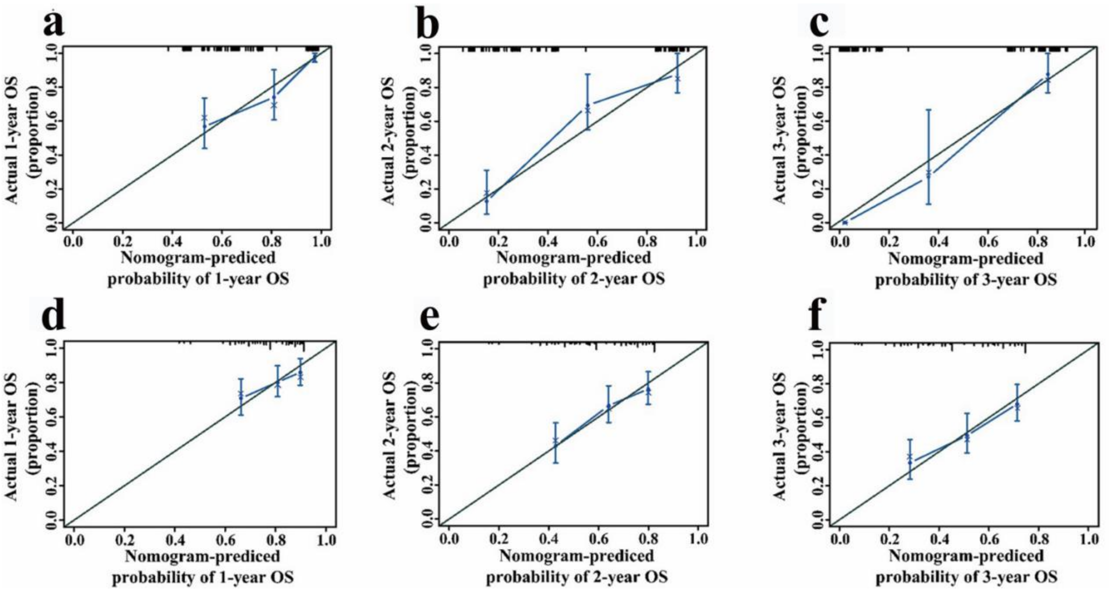

Fig. S6. Nomogram evaluation for predicting 1-, 2- and 3-year OS. (a-c) Calibration plots of the nomogram for predicting the probability of OS at 1 (a), 2 (b) and 3 years (c) in training cohort. (d-f) Calibration plots of the nomogram for predicting the probability of OS at 1 (d), 2 (e), 3 years (f) in meta-validation cohort.
